# DksA Modulates Antimicrobial Susceptibility of *Acinetobacter baumannii*

**DOI:** 10.3390/antibiotics10121472

**Published:** 2021-11-30

**Authors:** Nayeong Kim, Joo-Hee Son, Kyeongmin Kim, Hyo-Jeong Kim, Minsang Shin, Je-Chul Lee

**Affiliations:** Department of Microbiology, School of Medicine, Kyungpook National University, Daegu 41944, Korea; tbc02021@naver.com (N.K.); soun716@daum.net (J.-H.S.); horizon112@naver.com (K.K.); lo0729ve@naver.com (H.-J.K.); shinms@knu.ac.kr (M.S.)

**Keywords:** *Acinetobacter baumannii*, DksA, (p)ppGpp, antimicrobial susceptibility, efflux pump gene

## Abstract

The stringent response regulators, (p)ppGpp and DksA, modulate various genes involved in physiological processes, virulence, and antimicrobial resistance in pathogenic bacteria. This study investigated the role of DksA in the antimicrobial susceptibility of *Acinetobacter baumannii*. The ∆*dksA* mutant (KM0248D) of *A. baumannii* ATCC 17978 and its complemented strain (KM0248C) were used, in addition to the ∆*dksA* mutant strain (NY0298D) of clinical 1656-2 strain. The microdilution assay was used to determine the minimum inhibitory concentrations (MICs) of antimicrobial agents. Quantitative real-time PCR was performed to analyze the expression of genes associated with efflux pumps. The KM0248D strain exhibited an increase of MICs to quinolones and tetracyclines, whereas KM0248D and NY0298D strains exhibited a decrease of MICs to aminoglycosides. The expression of genes associated with efflux pumps, including *adeB*, *adeI/J*, *abeM*, and/or *tetA*, was upregulated in both ∆*dksA* mutant strains. The deletion of *dksA* altered bacterial morphology in the clinical 1656-2 strain. In conclusion, DksA modulates the antimicrobial susceptibility of *A. baumannii*. The ∆*dksA* mutant strains of *A. baumannii* upregulate efflux pump gene expression, whereas (p)ppGpp-deficient mutants downregulate efflux pump gene expression. (p)ppGpp and DksA conduct opposite roles in the antimicrobial susceptibility of *A. baumannii* via efflux pump gene regulation.

## 1. Introduction

*Acinetobacter baumannii* is a notorious nosocomial pathogen causing various infections, including pneumonia, bloodstream infections, and urinary tract infections, in critically ill patients [1,2]. *A. baumannii* rapidly acquired drug-resistant determinants, such as Ambler class B metallo-β-lactamase genes and class D *bla*_OXA_ genes, and the prevalence of carbapenem-resistant *A. baumannii* is a major concern worldwide [3,4]. In 2017, the World Health Organization proposed that carbapenem-resistant *A. baumannii* is the top priority pathogen for new antibiotic development [5]. Furthermore, under antibiotic selective pressure, this microorganism could develop resistance to commonly used antimicrobial agents by intrinsic resistance mechanisms, such as overexpression of efflux pump genes, permeability defects, and gene mutations that alter or modify target sites [3,6,7]. Of these resistance mechanisms, multiple efflux pumps play an important role in resistance to different classes of antimicrobial agents in *A. baumannii* [8,9,10]. The accumulation of acquired and intrinsic resistance mechanisms results in difficulty in the treatment of multidrug-resistant *A. baumannii* infections [11,12].

Bacterial alarmones, guanosine-5′,3′-tetraphosphate (ppGpp) and guanosine-5′,3′-pentaphosphate (pppGpp), collectively known as (p)ppGpp, are responsible for the bacterial stringent response by primarily regulating RNA polymerase (RNAP) activity [13,14]. DksA binds to the secondary channel of RNAP and allosterically modulates RNAP activity [15]. (p)ppGpp may work synergistically or independently with DksA [16]. The (p)ppGpp-deficient (Δ*rel*A Δ*spo*T) mutant was more susceptible to different classes of antimicrobial agents than the wild-type (WT) *Escherichia coli* strain [17]. Additionally, the ∆*dksA* mutant was more susceptible to antimicrobial agents, including β-lactams, aminoglycosides, quinolones, and tetracyclines, than the WT *E. coli* strain [18]. These results indicate that DksA and (p)ppGpp coordinately regulate the transcription of genes involved in antimicrobial resistance. There was no difference in the minimum inhibitory concentrations (MIC) of ciprofloxacin and ofloxacin between the WT and ∆*dksA* mutant strains of *Pseudomonas aeruginosa*, but the minimum bactericidal concentrations of quinolones increased in the ∆*dksA* mutants [19]. We recently demonstrated that (p)ppGpp-deficient (∆*A1S_0579*) mutant was more susceptible to antimicrobial agents, including cephalosporins, monobactam, carbapenems, fluoroquinolones, aminoglycosides, colistin, tetracyclines, and trimethoprim, than the WT *A. baumannii* ATCC 17978 strain via downregulation of various efflux pump genes [20]. However, the role of DksA in antimicrobial susceptibility has not been characterized in *A. baumannii*. This study investigated the role of DksA in the antimicrobial susceptibility of *A. baumannii* using WT *A. baumannii*, ∆*dksA* mutant, and *dksA*-complemented strains.

## 2. Results

### 2.1. The Effect of dksA on the Antimicrobial Susceptibility of A. baumannii ATCC 17978

**Figure 1:**
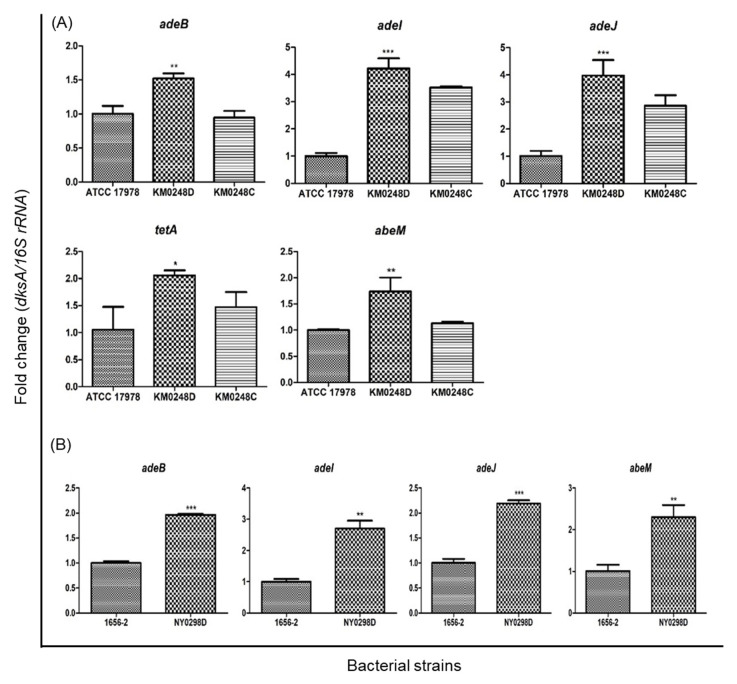
Expression of efflux pump genes in *A. baumannii* strains. (**A**) WT *A. baumannii* ATCC 17978, ∆*dksA* mutant (KM0248D), and *dksA*-complemented (KM0248C) strains were cultured in LB under shaking conditions for 18 h. (**B**) Clinical isolate 1656-2 and its ∆*dksA* mutant (NY0298D) strains were cultured in LB under shaking conditions for 18 h. Total RNA was extracted, and cDNA was synthesized. Gene expression was analyzed using qPCR. The data are presented as mean ± SD of three independent experiments. * *p* < 0.05, ** *p* < 0.01, *** *p* < 0.001 compared with the WT strain.

To examine the role of DksA in antimicrobial susceptibility of *A. baumannii* ATCC 17978, the minimum inhibitory concentrations (MICs) of antimicrobial agents for WT, ∆*dksA* mutant (KM0248D), and *dksA*-complemented (KM0248C) strains were determined. Of the 15 antimicrobial agents tested, MICs of five agents, including quinolones (nalidixic acid, ciprofloxacin, and levofloxacin) and tetracyclines (tetracycline and tigecycline) increased more than two-fold in the ∆*dksA* mutant strain compared with the WT strain (Table 1). However, the MICs of aminoglycosides (amikacin, gentamicin, and tobramycin) decreased more than two-fold in the ∆*dksA* mutant strain compared with the WT strain. Quantitative real-time PCR (qPCR) was conducted to determine whether efflux pump genes were responsible for the changes in the MICs of antimicrobial agents against the ∆*dksA* mutant strain. The expression of efflux pump genes, including *adeB*, *adeI*, and *adeJ* for resistance nodulation cell division (RND)-type multidrug efflux pumps, *tetA* for a major facilitator superfamily (MFS)-type drug efflux transporter, and *abeM* for a multidrug and toxic compound extrusion (MATE)-type multidrug efflux transporter, was significantly increased in the ∆*dksA* mutant strain compared with the WT *A. baumannii* ATCC 17978 strain (Figure 1A). However, the expression of the *adeI* and *adeJ* in the *dksA*-complemented strain was not restored compared to the WT strain.

### 2.2. The Effect of dksA on the Antimicrobial Susceptibility and Cellular Morphology of a Clinical A. baumannii Strain

**Figure 2:**
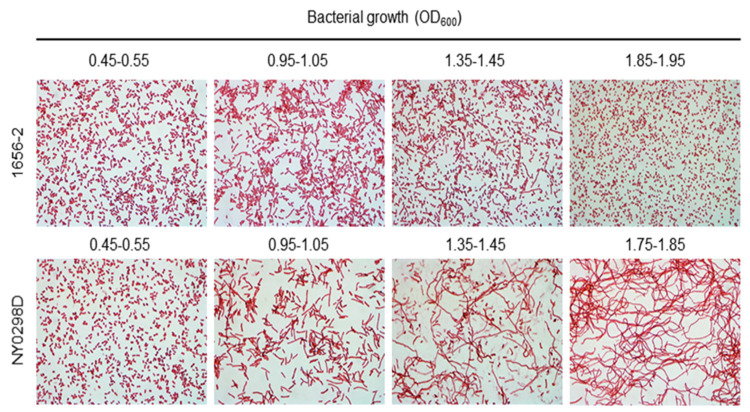
A morphological difference between *A. baumannii* 1656-2 and its ∆*dksA* mutant NY0298D strains. Bacteria were cultured in LB with shaking to reach the indicated OD_600_ and stained with Gram’s reagents. Bacterial morphology was observed using a light microscope. Magnification, 1000×.

To examine the role of DksA in the antimicrobial susceptibility and cellular morphology of a clinical *A. baumannii* strain, ∆*dksA* mutant (NY0298D) of the clinical 1656-2 strain was constructed (Supplementary Figure S1A). Deletion of *dksA* in *A. baumannii* 1656-2 was confirmed by PCR analysis (Supplementary Figure S1B). The expression of *dksA* was not observed in the ∆*dksA* mutant strain (Supplementary Figure S1C). Additionally, we determined whether *dksA* deletion changed the antimicrobial susceptibility of the clinical *A. baumannii* 1656-2 strain. No difference was observed in the MICs of quinolones and tetracyclines between WT and NY0298D strains. However, the NY0298D strain exhibited increased susceptibility to aminoglycosides (amikacin, gentamicin, and tobramycin) like the ∆*dksA* mutant strain of *A. baumannii* ATCC 17978 (Table 1). The expression of efflux pump genes, including *adeB*, *adeI*, *adeJ*, and *abeM*, significantly increased in the ∆*dksA* mutant strain, compared with that in the WT strain (Figure 1B). The ∆*dksA* mutant NY0298D strain displayed more morphological heterogeneity than the WT strain at an optical density of 600 nm (OD_600_) of 0.95–1.05 to 1.75–1.85 (Figure 2). These results suggest that *dksA* deletion in the clinical 1656-2 strain increases efflux pump gene expression and alters bacterial morphology.

## 3. Discussion

The (p)ppGpp-deficient and ∆*dksA* mutants of *E. coli* exhibit increased susceptibility to antimicrobial agents [17,18], implying that (p)ppGpp and DksA contribute to antimicrobial resistance in *E. coli*. The (p)ppGpp-deficient mutant of *A. baumannii* ATCC 17978 also exhibited increased susceptibility to antimicrobial agents [20]. However, in the present study, ∆*dksA* mutant of *A. baumannii* ATCC 17978 exhibited decreased susceptibility to quinolones and tetracyclines, whereas ∆*dksA* mutants of *A. baumannii* ATCC 17978 and 1656-2 exhibited increased susceptibility to aminoglycosides.

The deletion of *dksA* upregulated the expression of *adeB*, *adeI, adeJ*, *abeM* and/or *tetA* in *A. baumannii* ATCC 17978 and the clinical 1656-2 strain. *A. baumannii* ATCC 17978 was susceptible to quinolones and tetracyclines, whereas the clinical 1656-2 strain was resistant to quinolones and tetracycline and susceptible to tigecycline [21]. In the 1656-2 strain, resistance to quinolones was mediated by the mutations in the quinolone-resistance determining region of *gyrA*, and resistance to tetracyclines was potentially mediated by several efflux pump genes [22]. Furthermore, multidrug-resistant *A. baumannii* strains decrease cell envelope permeability against antimicrobial agents [23]. Therefore, the upregulation of efflux pump genes directly contributed to increased MICs of quinolones and tetracyclines in the ∆*dksA* mutant of ATCC 17978, although efflux pump gene upregulation could not change the MICs of quinolones and tetracyclines in ∆*dksA* mutant of 1656-2. Both ∆*dksA* mutants of ATCC 17978 and 1656-2 were more susceptible to aminoglycosides than the WT strains. In a previous study, the (p)ppGpp-deficient strain of *A. baumannii* ATCC 17978 was more susceptible to aminoglycosides than the WT strain [20]. Because (p)ppGpp and DksA inhibit the transcription of genes involved in the synthesis of translational machinery during the stringent response or stressful conditions [15,24], (p)ppGpp-deficient and ∆*dksA* mutants cannot inhibit the transcription of ribosomal genes, potentially increasing susceptibility to aminoglycosides. Combined with the previous results, the present study demonstrates that (p)ppGpp and DksA play an opposing role in the regulation of genes associated with efflux pumps. Further studies would be required to understand the regulatory mechanisms of multiple genes linked with intrinsic resistance by DksA and (p)ppGpp in *A. baumannii*.

The present study demonstrated that ∆*dksA* mutants of clinical 1656-2 exhibited more morphological heterogeneity than the WT strain. Previous studies have reported that ∆*dksA* mutant and (p)ppGpp-deficient mutant strains exhibited more morphological heterogeneity than the WT *A. baumannii* ATCC 17978 strain [20,25]. The (p)ppGpp-deficient and ∆*dksA* mutants in *E. coli* also exhibited a more filamentous morphology than the WT strain [16]. These results indicate that (p)ppGpp and DksA coordinately regulate genes associated with cellular morphology or cell division.

This study demonstrates that *dksA* deletion upregulates efflux pump gene expression in *A. baumannii* strains. However, (p)ppGpp deficiency downregulates the expression of efflux pump genes in *A. baumannii* [16]. Overall, RNAP-binding global regulators (p)ppGpp and DksA can modulate antimicrobial susceptibility in *A. baumannii*, but they play opposite roles in antimicrobial resistance through regulating the efflux pump genes.

## 4. Materials and Methods

### 4.1. Bacterial Strains

Bacteria, including WT, ∆*dksA* mutant, and *dksA*-complemented strains, and plasmids used in this study are listed in Table 2. *A. baumannii* and *E. coli* strains were cultured in lysogeny broth (LB) (BioShop, Burlington, ON, Canada) at 37 °C. Mutant strains were selected in LB media containing chloramphenicol (20 μg/mL) or erythromycin (30 μg/mL).

### 4.2. Construction of the ∆dksA Mutant of 1656-2 Strain

The ∆*ABK1_0298* gene of clinical *A. baumannii* 1656-2 strain, corresponding to the *A1S_0248* gene of *A. baumannii* ATCC 17978, was deleted by a markerless gene deletion method [27]. Genomic DNAs purified from *A. baumannii* 1656-2 and pFL02 were used as polymerase chain reaction templates for the amplification of *dksA* and erythromycin resistance cassettes, respectively. The upstream and downstream regions of *dksA* were combined with an erythromycin resistance cassette through overlap extension PCR using specific primers with a ProFlex PCR system (Applied Biosystems, Foster City, CA, USA) (Supplementary Table S1). This mutated DNA fragment was ligated into *Apa*I-digested pDM4. The pDM4 carrying the mutated DNA fragment was inserted into the chromosome of *A. baumannii* 1656-2 strain by transformation using Gene Pulser Xcell (Bio-Rad, Hercules, CA, USA) and homologous recombination (Supplementary Figure S1A). The ∆*dksA* mutant of *A. baumannii* 1656-2 was named NY0298D (Table 2).

### 4.3. Antimicrobial Susceptibility Testing

The MICs of antimicrobial agents were determined by the microdilution method according to the Clinical Laboratory Standards Institute (CLSI) [28]. Antimicrobial agents included aminoglycosides (amikacin, gentamicin, and tobramycin), carbapenems (imipenem and meropenem), cephalosporins (ceftazidime, cefoxitin, and cefotaxime), quinolones (nalidixic acid, ciprofloxacin, and levofloxacin), tetracyclines (tetracycline and tigecycline), colistin and trimethoprim. *E. coli* ATCC 25922 and *Pseudomonas aeruginosa* ATCC 27853 were used as quality control strains.

### 4.4. RNA Isolation and qPCR

Bacteria were cultured in LB under shaking conditions for 18 h to analyze the efflux pump gene expression. Total RNA was extracted using the RNeasy Mini Kit (Qiagen, Valencia, CA, USA). Reverse transcription was conducted to synthesize cDNA using 1.5 μg of total RNA, random hexamer primers, and TOPscript reverse transcriptase (Enzynomics, Daejeon, Korea). The specific primers for efflux pump genes are listed in Supplementary Table S2. Gene transcripts were quantified using TOPreal qPCR 2Χ PreMIX (SYBR Green with high ROX) (Enzynomics) with a StepOnePlus Real-Time PCR Systems (Applied Biosystems). Melting curve analysis was conducted to evaluate the amplification specificity. The expression of efflux pump genes was normalized to the expression of the 16S rRNA gene, and the fold change was determined. Gene expression assays were performed in three independent experiments.

### 4.5. Gram Staining

*A. baumannii* strains were cultured overnight before being diluted to an OD_600_ of 1.0. The bacterial samples were diluted 1:20 in fresh LB and cultured in LB under shaking conditions to reach the indicated OD_600_. Bacteria were stained by Gram reagents (YD Diagnotics, Gyeonggi, Korea) [29] and then observed under a Nikon Eclipse E600 microscope (Nikon, Tokyo, Japan).

### 4.6. Statistical Analysis

Data were analyzed using GraphPad Prism 5.0 software (San Diego, CA, USA). Data from different experimental groups were analyzed using one-way ANOVA with Dunnett’s post hoc analysis or Student’s t-test. Differences of *p* < 0.05 were considered statistically significant.

## Figures and Tables

**Table 1:** Antimicrobial susceptibility of wild-type *A. baumannii*, ∆*dksA* mutant, and *dksA*-complemented strains.

Antibacterial Agents	MIC (μg/mL)			Fold Change (KM0248D/WT)	MIC (μg/mL)		Fold Change (NY0298D/WT)
	ATCC 17978	KM0248D	KM0248C		1656-2	NY0298D	
Nalidixic acid	4	16	8	4	>256	>256	1
Ciprofloxacin	0.125	0.5	0.125	4	64	64	1
Levofloxacin	0.063	0.125	0.063	2	16	16	1
Cefoxitin	128	128	128	1	>256	>256	1
Cefotaxime	16	16	16	1	>256	>256	1
Ceftazidime	4	4	4	1	>256	>256	1
Imipenem	0.125	0.125	0.125	1	16	16	1
Meropenem	0.25	0.25	0.25	1	32	32	1
Amikacin	1.0	0.25	0.5	0.25	64	32	0.5
Gentamicin	0.5	0.25	0.5	0.5	256	128	0.5
Tobramycin	0.5	0.125	0.25	0.25	128	64	0.5
Tetracycline	1	2	2	2	32	32	1
Tigecycline	0.125	0.5	0.125	4	1	1	1
Colistin	2	2	2	1	2	2	1
Trimethoprim	>32	>32	>32	1	32	32	1

**Table 2:** Bacterial strains and plasmids used in this study.

Bacteria/Plasmids	Relevant Characteristics	Reference of Source
*A. baumannii*		
ATCC 17978	Wild-type strain	ATCC
KM0248D	∆*A1S_0248* of *A. baumannii* ATCC 17978	[25]
KM0248C	*A1S_0248* with T1 terminator in KM0248D	[25]
1656-2	Clinical isolate	[21]
NY0298D	∆*ABK1_0298* of *A. baumannii* 1656-2	This study
Plasmids		
pDM4	Suicide vector, *ori* R6K; Cm^r^; sacB	GenBank accession no. KC795686
pFL02	pWH1266 with *armA* coding region and its promoter less *nptI*, and origin of replication with *ermAM*; Km^r^, Ery^r^	[26]

Abbreviations: Cm^r^, chloramphenicol-resistant; Km^r^, Kanamycin-resistant; Ery^r^, erythromycin-resistant.

## Data Availability

The authors confirm that the data supporting the findings of this study are available within the article and its Supplementary Materials.

## Supplementary Materials

The following are available online at https://www.mdpi.com/article/10.3390/antibiotics10121472/s1, Figure S1: Construction of the ∆*dksA* mutant strain, Table S1: Primers used for the DNA cloning in this study, Table S2: Primers used for qPCR in this study.
