# Supplementary material for: DksA Modulates Antimicrobial Susceptibility of Acinetobacter baumannii
Source: Antibiotics (Basel). 2021 Nov 30;10(12):1472. doi: 10.3390/antibiotics10121472 (PMC8698368; doi:10.3390/antibiotics10121472)
Supplement: Supplementary file 1 [file antibiotics-10-01472-s001.zip › antibiotics-1461032-supplementary.pdf]

Supplementary Materials

Supplementary Table S1. Primers used for the DNA cloning in this study.

Primer	Sequence (5`→3`)*
ABK1_0298_UP_ApaI_F	GTTGGGCCCCCGAAGAGGTAGGACGGTGAC
ABK1_0298_UP_R	CTTAACCGTCACAATTACATAATAGGGCATTCCTCATCATACACGTATTATC
ABK1_0298_down_16_F	AATGCCCTATTATGTAATTGTGACGGTTAAGAATTCTCCAG
ABK1_0298_down_16_R	CAAGTCAGCACGAACACGAATCAAACTTCTAAACCATGAAGCGTTAT
EryR_F	TCGTGTTCGTGCTGACTTG
EryR_ApaI_R	GTTGGGCCCGACCTCTTTAGCTCCTTGGAAGC

* Underlined sequences indicate regions that are not complementary to the templates.

Supplementary Table S2. Primers used for qPCR in this study.

Primers	Sequence (5´ to 3´)	Target genes
16S rRNA-F	GCACAAGCGGTGGAGCAT	16S rRNA for ATCC 17978 and 1656-2
16S rRNA-R	CGAAGGCACCAATCCATCTC	
DksA-F	TCTTGGGTAGCACGGTCATTT	*dksA* for ATCC 17978 and 1656-2
DksA-R	GGAAGGACAGCTCGAGCATT	
AdeB-F	GCACAACCAGCATCACAGAAA	*adeB* for ATCC 17978
AdeB-R	AAAAGCAGTCTGAATCACAAATGG	
AdeB-F	CCTTGTGGCAACCCTTCATT	*adeB* for 1656-2
AdeB-R	CCTGCTTTACTGGCTGCTCAA	
AdeI-F	TGGTTATTCTACAATTCGCTCTCCTAT	*adeI* for ATCC 17978 and 1656-2
AdeI-R	CAAAGCACCAGCCGTTACTG	
AdeJ-F	GCGGGCAGCCGTATGA	*adeJ* for ATCC 17978 and 1656-2
AdeJ-R	ACGCCGAGAATGGAACCA	
TetA-F	GGCAAAAATCATCCAACCACTT	*tetA* for ATCC 17978
TetA-R	CGTGCTAATCGGTATTGCTTGTT	
AbeM-F	GCCCAGTTCTTTTCGCCATA	*abeM* for ATCC 17978
AbeM-R	CCACTTTCTCTTGCCATTGCT	
AbeM-F	GAAGCCCAGTTCTTTTCACCATA	*abeM* for 1656-2
AbeM-R	CCACTTTCTCTTGCCATTGCT	

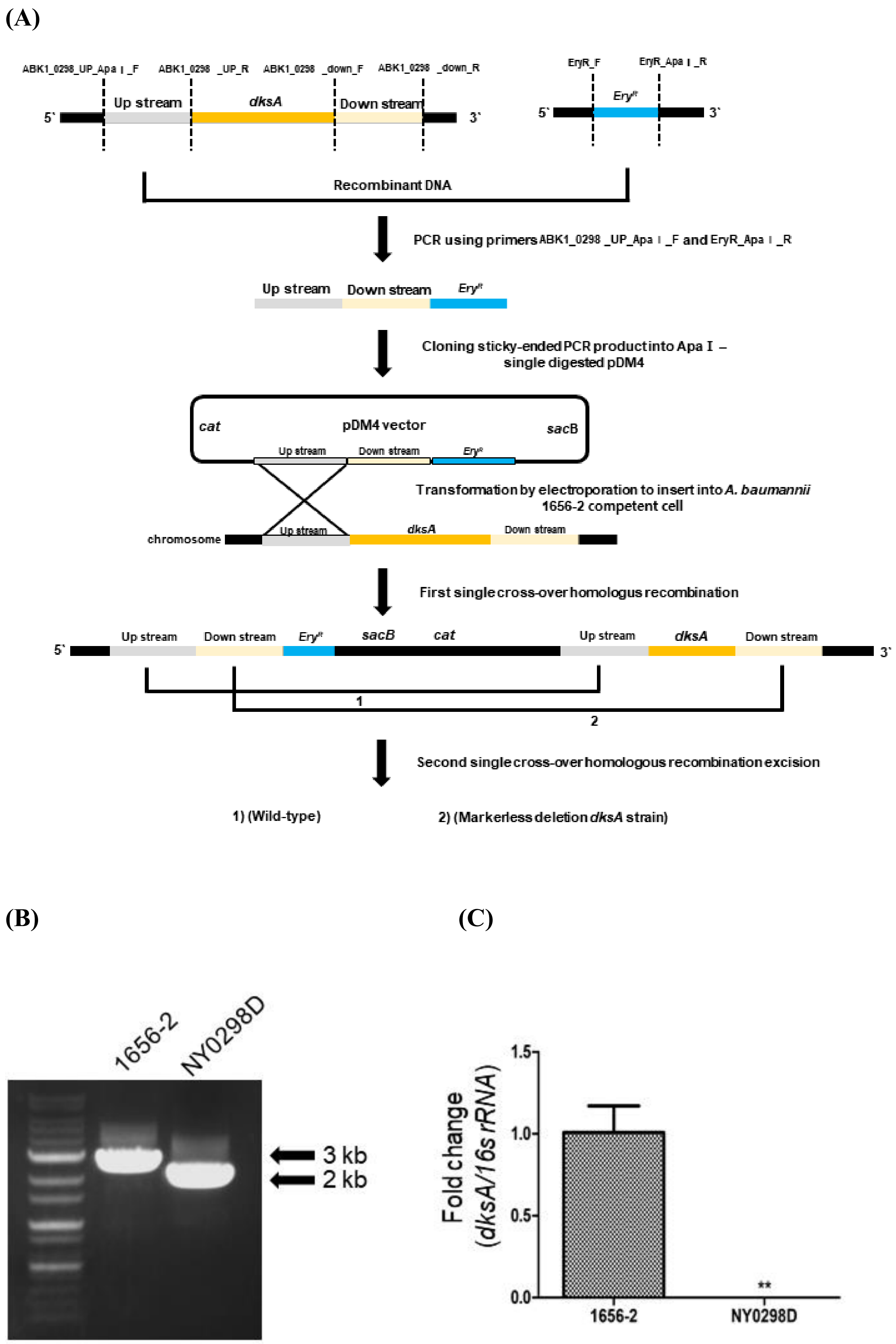
(A)
ABK1_0298_UP_Apa I _F
ABK1_0298 _UP_R
ABK1_0298 _down_F
ABK1_0298 _down_R
Up stream
dksA
Down stream
5`
3`
EryR_F
EryR_Apa I _R
Ery^R
Recombinant DNA
PCR using primers ABK1_0298 _UP_Apa I _F and EryR_Apa I _R
Cloning sticky-ended PCR product into Apa I – single digested pDM4
cat
pDM4 vector
sacB
Transformation by electroporation to insert into A. baumannii 1656-2 competent cell
chromosome
First single cross-over homologus recombination
1
2
Second single cross-over homologous recombination excision
1) (Wild-type)
2) (Markerless deletion dksA strain)
(B)
1656-2
NY0298D
3 kb
2 kb
(C)
Fold change (dksA/16s rRNA)
1.5
1.0
0.5
0.0
**
1656-2
NY0298D

Supplementary Figure S1. Construction of the Δ*dksA* mutant strain. (**A**) Construction of Δ*dksA* mutant (NY0298D) in *A. baumannii* 1656-2. (**B**) The deletion of *dksA* in the NY0298D strain was confirmed by PCR using the primers ABK1_0298_UP_ApaI_F and ABK1_0298_down_16_R (Supplementary Table S1). The WT WT 1656-2 and NY0298D strains had amplicon sizes of 2,601 bp and 2,070 bp, respectively. (**C**) The expression of *dksA* was determined in the WT and Δ*dksA* mutant strains using qPCR. The data are presented as mean ± SD of three independent experiments. ** p <0.005 compared to WT strain.
